# Hypertension and obesity independently drive hypertrophy and alter mitochondrial metabolism in a mouse model of heart failure with preserved ejection fraction

**DOI:** 10.14814/phy2.70072

**Published:** 2024-09-27

**Authors:** Benjamin Werbner, Sophie L. Stephens, Deborah Stuart, Travis M. Hotchkiss, Jonathan Chapman, Katsuhiko Funai, Nirupama Ramkumar, Sihem Boudina

**Affiliations:** ^1^ Department of Nutrition and Integrative Physiology University of Utah Salt Lake City Utah USA; ^2^ Division of Nephrology and Hypertension University of Utah School of Medicine Salt Lake City Utah USA

**Keywords:** cardiac hypertrophy, chronic hypertension, fatty‐acid oxidation, HFpEF, obesity

## Abstract

Heart failure with preserved ejection fraction (HFpEF) has recently emerged as an insidiously and increasingly prevalent heart failure phenotype. HFpEF often occurs in the context of hypertension and obesity and presents with diastolic dysfunction, ventricular hypertrophy, and myocardial fibrosis. Despite growing study of HFpEF, the causal links between early metabolic changes, bioenergetic perturbations, and cardiac structural remodeling remain unclear. This study sought to elucidate the contribution of the respective pathophysiological drivers of the HFpEF symptom suite using a recently developed two‐hit mouse model. By studying the independent and concomitant consequences of hypertension and obesity‐driven metabolic dysfunction on cardiac structure and function, we revealed the causative drivers of cardiac functional, structural, and metabolic remodeling in male HFpEF mice. We found that hypertensive male mice developed diastolic dysfunction and cardiac hypertrophy regardless of obesity status and that obese mice exhibited altered systemic glucose metabolism and increased cardiac mitochondrial fatty‐acid metabolism independent of hypertension status. Taken together, our results suggest that the cardiac structural and metabolic HFpEF symptoms in this two‐hit model occur as direct results of each of the two “hits.” The results of this study help to clarify the pathogenic HFpEF cascade, providing causal insights that may aid in the development of more precisely targeted therapeutics.

## 1 INTRODUCTION

Heart disease is the leading cause of death for both men and women in the United States, with total healthcare costs in the United States exceeding $230 billion annually according to the Centers for Disease Control and Prevention. Common risk factors for heart disease include age, hypertension, obesity, diabetes, hyperlipidemia, and inactive lifestyle, all of which are on the rise over the last several decades. Heart failure with preserved ejection fraction (HFpEF) has recently emerged as an insidiously and increasingly prevalent heart failure phenotype now accounting for over 50% of heart failure mortality (Dunlay et al., 2017; Tsao et al., 2018). HFpEF often occurs in the context of hypertension and systemic metabolic dysregulation and presents with diastolic dysfunction, ventricular hypertrophy, and myocardial fibrosis (Abudureyimu et al., 2022; Withaar et al., 2022).

While cardiac structural remodeling and bioenergetic changes are two well‐known hallmarks of advanced heart disease, the mechanistic and temporal links between early metabolic changes, bioenergetics perturbations, and cardiac structural remodeling in HFpEF remain unclear (Ritchie & Abel, 2020). In fact, there is considerable disagreement in the current literature regarding cardiac fuel utilization in HFpEF, with several groups reporting decreased mitochondrial fatty acid oxidation (Hahn et al., 2023; Tong et al., 2021) and others reporting increased mitochondrial utilization of fatty acids to compensate for diminished glucose availability and pyruvate oxidation (Guven et al., 2024; Sun et al., 2024). Thus, the current study sought to elucidate the causal interplay of cardiac mitochondrial metabolism and myocardial structural remodeling using a recently developed two‐hit mouse model of HFpEF (Schiattarella et al., 2019) to determine the causative drivers and downstream consequences of the pathogenic HFpEF cascade. A deeper understanding of the temporal and causal cascades in the early pathogenesis of HFpEF may present attractive therapeutic options for early intervention, which would greatly benefit a growing population of HFpEF patients with of yet very limited treatment options (Abudureyimu et al., 2022; Withaar et al., 2022).

A mouse model of HFpEF mimicking the human phenotype was developed by a group at UT Southwestern and involves the administration of the pan nitric oxide synthase inhibitor nitro‐L‐arginine methylester (L‐NAME or LN) in the drinking water of adult, male mice in combination with high‐fat diet feeding (HF) (Schiattarella et al., 2019). By inhibiting nitric oxide synthase, L‐NAME impairs systemic vasodilation, increasing vascular resistance and blood pressure (Bennett & Gardiner, 1992). This presents a mechanical challenge to the heart muscle, which is simultaneously challenged metabolically by prolonged high‐fat feeding. In addition to recapitulating the two most common human risk factors (hypertension and obesity), the resulting cardiac phenotype was validated to mimic the human phenotype in male, but not female mice; however, the relative contribution of each risk factor to the HFpEF symptom suite was not investigated. Previous literature suggests that increased mechanical demand on the heart resulting from systemic hypertension likely drives concentric cardiac hypertrophy, whereas prolonged systemic metabolic dysregulation and lipotoxicity have been linked with alterations in heart bioenergetics and mitochondrial function (Werbner et al., 2023). Thus, we hypothesized that hypertension is the primary driver of cardiac structural remodeling while obesity drives metabolic changes in the heart.

We found that all chronically hypertensive male mice, regardless of obesity status, presented with whole‐heart and cardiomyocyte hypertrophy, grade 3 diastolic dysfunction, and preserved systolic function. On the other hand, chronic high‐fat feeding, regardless of hypertension status, resulted in increased cardiac mitochondrial fatty‐acid oxidation capacity, which was substantiated by transcript and protein levels of fatty‐acid transporters and oxidation enzymes. Obese and glucose‐intolerant hypertensive mice appear to maintain systolic function against increased vascular resistance and ventricular filling pressures by increasing cardiac muscle mass and mitochondrial bioenergetic capacity, particularly utilization of fatty‐acid substrates. Taken together, these results shed light on the causal contribution of two primary HFpEF risk factors that may aid in the development of more precisely targeted therapeutics in lean and obese hypertensive patients during the early progression to HFpEF.

## 2 MATERIALS AND METHODS

### 2.1 Animal care and treatment

Animal studies were approved by the University of Utah Institutional Animal Care and Use Committee. All procedures were carried out according to the NIH Guide for the Care and Use of Laboratory Animals. Four groups of 12‐week‐old male C57BL/6J mice (Jackson Labs) were housed 4–5 per cage on a 12‐h light dark cycle and provided ad libitum access to the following food and water combinations: normal chow and drinking water (NC; Inotiv Teklad 2920X: 6% kcal fat, 19% kcal protein, 3 kcal/g), normal chow and 0.5 mg/mL L‐NAME (APExBIO, A7088) drinking water (NC + LN), 60% high‐fat chow and normal drinking water (HF; Inotiv TD.06414: 60% kcal fat, 18% kcal protein, 5 kcal/g), or high‐fat chow and 0.5 mg/mL L‐NAME drinking water (HF + LN) for a duration of 18 weeks. See Data S1 for additional details.

### 2.2 Body composition and systemic metabolic phenotyping

See Data S1.

### 2.3 Echocardiography

Mice were induced and maintained under light anesthesia by inhalation of isoflurane. Cardiac function was measured using a VisualSonics Vevo 2100 ultrasound machine with MS400 probe. B‐ and M‐mode sequences were acquired in the parasternal long‐ and short‐axis orientations and used to assess systolic function and to quantify morphological features. Mitral inflow velocities (E and A), and early diastolic mitral annulus tissue velocity (e') were measured in the apical, four‐chamber view using flow and tissue Doppler, respectively, and used to assess diastolic function on the basis of E/A and E/e' ratios. Echocardiographic data was analyzed using VisualSonics VevoLab.

### 2.4 Blood pressure telemetry

A subset of *n* = 4 randomly selected mice from each treatment group were implanted with blood pressure telemetry sensors (TA11‐PAC10, Data Sciences International) in the carotid artery, just above the intersection with the subclavian artery. Blood pressure was measured continuously over two adjacent 24‐h periods by serially sampling the mean arterial pressure at 10‐min intervals. Mean arterial pressures from the two 12‐h dark periods were averaged and reported as such. Given the somewhat invasive nature of the telemetry measurement, no additional experiments were performed on mice or tissues from mice after implantation of telemetry devices; thus, only a randomly selected subset of mice from each treatment group had blood pressure measured telemetrically.

### 2.5 Mitochondrial isolation and quantification of mitochondrial oxygen consumption rate

Approximately 80 mg of fresh left‐ventricular tissue was homogenized in cold, mannitol‐based isolation buffer (MIB) and differentially centrifugated to obtain an enriched fraction of cardiac mitochondria that were resuspended at approximately 2 μg/μL of protein in MIB. 5 μg/mL of isolated mitochondrial protein was loaded into an Oroboros Oxygraph‐2k chamber in KCl‐based mitochondrial respiration buffer (Buffer Z) and allowed to equilibrate before addition of one of the following substrate combinations: 2.5 mM malate (PHR1273) and 12.5 μM palmitoyl‐carnitine (P1645), 2.5 mM malate and 5 mM pyruvate (P2256), 2.5 mM malate and 5 mM α‐ketoglutarate (75890), or 5 mM succinate (S2378) and 0.5 μM rotenone (R8875) (Tomar et al., 2022). After stabilization, 150 μM adenosine diphosphate or ADP (A5285) was added to stimulate State 3 respiration. In at least one additional sample from each group, exogenous cytochrome c (C7752) was added during State 3 to assess mitochondrial membrane integrity; respiration did not increase more than 8% after cytochrome c addition, suggesting intact outer mitochondrial membranes. All respiratory substrates were acquired from Sigma Aldrich and are identified by catalog number above. The dissolved oxygen concentration remained above 100 μM for the duration of all experiments. Respirometry traces were analyzed with Orobros Datlab software to calculate the rate of oxygen consumption (*j*O_2_).

### 2.6 Quantification of mitochondrial ATP production rate

Isolated mitochondrial suspensions were loaded in identical conditions as above and allowed to equilibrate for 5 min. After stabilization, one of the following substrate combinations was added: 2.5 mM malate and 12.5 μM palmitoyl‐L‐carnitine or 2.5 mM malate and 5 mM pyruvate. After 2 min of equilibration, 150 μM ADP was added to stimulate State 3 respiration. After 2 min of equilibration, 5 μL of the respiration media was sampled every 10 s for 2 min and immediately diluted to 1:100 in ice‐cold ultra‐pure distilled water. This time‐series was analyzed by luciferin–luciferase luminescence assay (Thermo Fisher, A22066) and used to calculate the rate of ATP production (*j*ATP). ATP/O ratio for each sample was determined by dividing *j*ATP by *j*O_2_ and dividing the result by two.

### 2.7 Histology

Transverse heart sections were acquired after sacrifice and fixed for 72 h in 10% neutral‐buffered formalin before being embedded in paraffin, sectioned to 5 μm thickness, and mounted on glass microscopy slides. Sections were deparaffinized and stained with wheat germ agglutin or WGA (Thermo Fisher, W11261) conjugated to Alexa Fluor 488 and 4′,6‐diamidino‐2‐phenylindole or DAPI (Sigma Aldrich, D9542) to assess cardiomyocyte cross‐sectional area. Sections were imaged at 40× magnification at excitation/emission wavelengths of 499/420 nm, with five non‐overlapping images per heart. Only mid‐cell, transversely sectioned cardiomyocytes containing nuclei visibly stained with DAPI were analyzed for cross‐sectional area within each image. See Data S1 for additional details.

### 2.8 Western blotting

Based on the results of our isolated mitochondrial respiration experiments, select protein levels were assessed via Western blotting. See Data S1 for details. Given the limited size of loading gels and the importance of loading samples on the same gel/membrane for consistent quantitative comparisons, only a subset of *n* = 6/group randomly selected samples were immunoblotted.

### 2.9 Gene expression analysis

See Data S1.

### 2.10 Statistical analysis

All group measurements were analyzed for statistically significant differences by first applying a two‐way analysis of variance (ANOVA) in GraphPad Prism, using diet (NC or HF) and drug treatment (LN+ or LN−) as the two factors. Sidak's multiple comparisons post‐hoc analysis was conducted to determine pairwise significant differences between groups only for the factors found significant in the two‐way ANOVA (*p* < 0.05). All *p*‐values from pairwise comparisons are reported as multiplicity‐adjusted *p*‐values. See Data S1 for additional details.

## 3 RESULTS

### 3.1 Fat‐fed mice developed obesity and glucose intolerance

Both groups of fat‐fed mice exhibited 45% increases in body weight versus chow‐fed controls after 18 weeks of treatment, and nuclear magnetic resonance spectroscopy revealed that HF groups had 140% more relative fat mass than chow‐fed mice (*p* < 0.001) (Figure S1A,B). L‐NAME administration did not affect body weight or body composition at the termination of treatment (*p* = 0.197). Both groups of fat‐fed mice exhibited fasting hyperglycemia (40% increase), fasting hyperinsulinemia (55% increase), delayed glucose homeostasis kinetics (35%–80% increased AUC), and glucose‐stimulated insulin levels (40% increase) versus normal chow controls when administered intraperitoneal glucose (all *p* < 0.001; Figure S1C–G). Both groups of normal chow‐fed mice maintained glucose homeostasis as well as fasting and fed insulin levels. Based on the glucose area under the curve, LN moderated glucose homeostasis kinetics after HF‐feeding (Figure S1F,G; *p* < 0.001), however assessment of fasting‐ and glucose‐challenged insulin levels suggests that this effect may not be solely mediated by insulin, as insulin levels were not different between the two fat‐fed groups (Figure S1D,E; *p* > 0.35). This is substantiated by previous literature which suggests that L‐NAME may have a mild glucose‐sensitizing effect only in fat‐fed mice (Tsuchiya et al., 2007).

### 3.2 L‐NAME‐treated mice developed hypertension, diastolic dysfunction, and cardiac hypertrophy

**FIGURE 1:**
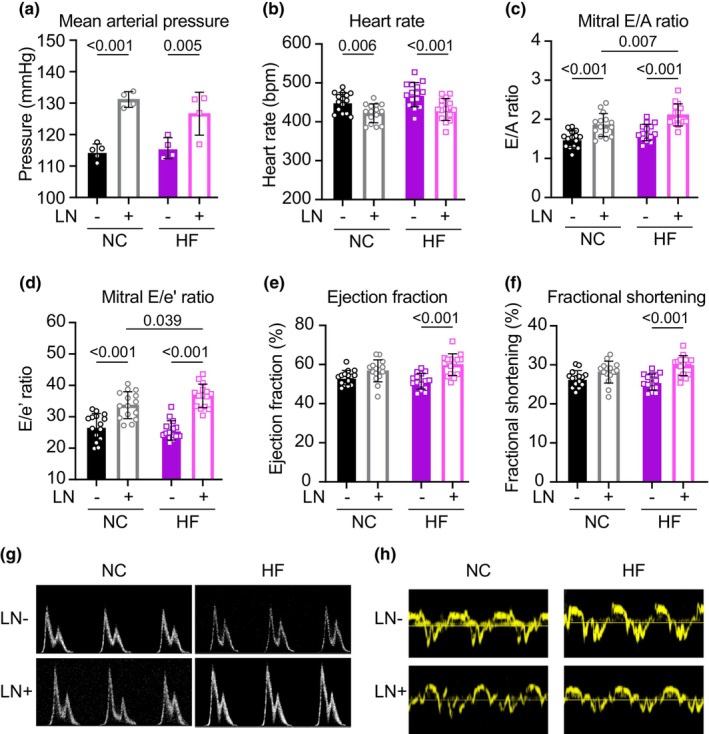
(a) Mice receiving LN exhibited significant increases in 12‐h (dark hours) average mean arterial pressure measured telemetrically. (b) Mice receiving LN exhibited decreased heart rates (measured via echocardiography), consistent with the baroreceptor reflex. (c, d) Both groups receiving LN exhibited elevated mitral E/A and E/e' ratios. (e, f) HFLN+ mice exhibited a mild hyper‐contractile phenotype after 18 weeks of treatment. (g, h) Representative mitral inflow and tissue annulus Doppler traces indicate the development of grade 3 diastolic dysfunction in hypertensive mice. Panel (a): *N* = 4–5/group; (b–f): *N* = 15/group. As for all figures, pairwise significant differences were determined with Sidak's multiple comparison post‐hoc analysis performed only on factors determined to be significant (*p* < 0.05) via two‐way ANOVA.

Both groups of mice receiving L‐NAME (LN+) exhibited 17 mmHg increases in average mean arterial pressure measured telemetrically over the 12‐h dark period (12% increase vs. LN‐, *p* < 0.005), with the same extent of blood pressure elevation observed in both groups receiving L‐NAME regardless of diet (Figure 1a). Both groups of hypertensive mice, regardless of diet, exhibited 6%–8% decreases in heart rate versus controls (measured by echocardiography), consistent with the baroreceptor reflex (Figure 1b; *p* < 0.006).

Both NC and HF groups receiving L‐NAME exhibited 25% increases in mitral inflow and mitral annulus tissue velocity ratios (E/A and E/e', respectively) versus controls after 18 weeks of treatment (*p* < 0.001; Figure 1c,d), indicating increased filling pressures and impaired ventricular relaxation in both groups with elevated blood pressure. Additionally, the HFLN+ group exhibited 14% and 9% increases in E/A and E/e' ratios respectively compared to the NCLN+ group (*p* < 0.04), indicating an additive effect of the two model hits on diastolic function. Quantitatively, this diastolic dysfunction was driven by both increased early diastolic filling velocity (increased E), decreased late diastolic filling velocity (decreased A), and decreased early mitral tissue velocity (decreased e'), consistent with Grade 3 diastolic dysfunction (Figure 1g,h). While no significant changes were observed in the systolic function of mice receiving only LN or only HF, the HFLN+ group exhibited a 16% increase in ejection fraction and fractional shortening (*p* < 0.001; Figure 1e,f).

**FIGURE 2:**
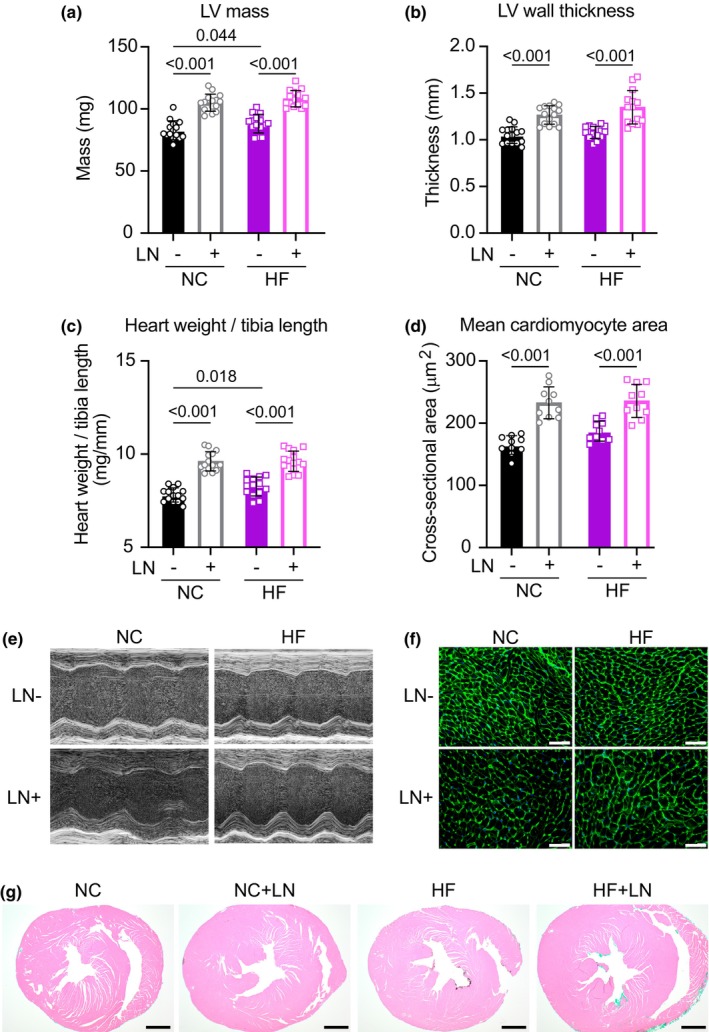
Both hypertensive groups developed cardiac hypertrophy after 18 weeks of treatment, assessed by echocardiography (a, b, e), heart weight normalized to tibia length (c), average left‐ventricular cardiomyocyte cross‐sectional area (d, f; scale bars = 50 μm), and representative transverse cardiac cross‐sections stained with hematoxylin and eosin (g; scale bars = 200 μm). Panels (a–c): *N* = 15/group; (d): *N* = 10/group.

Morphologically, both NC and HF groups receiving L‐NAME exhibited 25% increases in left‐ventricular mass and left‐ventricular posterior wall thickness versus controls (measured by echocardiography; *p* < 0.001; Figure 2a,b,e), indicating a similar extent of gross ventricular hypertrophy associated with similar elevations in blood pressure. Corroborating this in vivo assessment of cardiac morphology, all mice receiving L‐NAME exhibited 15%–20% increases in heart weight normalized to tibia length and left‐ventricular cardiomyocyte cross‐sectional area assessed by WGA staining (*p* < 0.001; Figure 2c,d,f). Representative images of transverse whole‐heart sections stained with hematoxylin and eosin are provided in Figure 2g.

### 3.3 Fat‐fed mice exhibit increased cardiac mitochondrial fatty‐acid oxidation capacity

**FIGURE 3:**
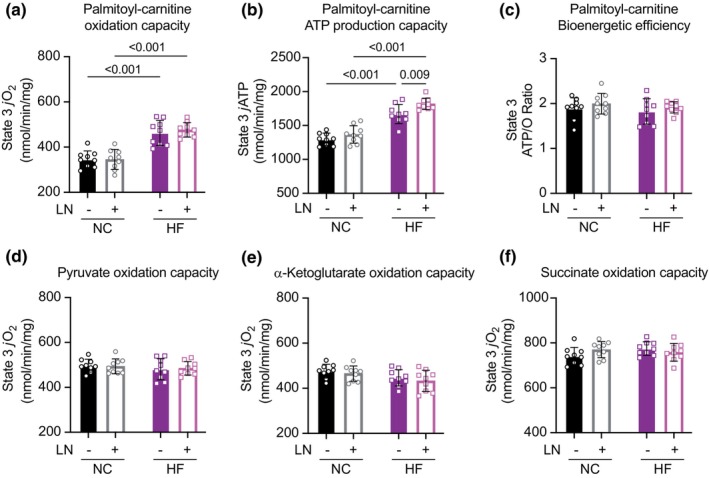
Isolated cardiac mitochondria from fat‐fed mice exhibited significantly increased fatty‐acid oxidation capacity (a) without net change in bioenergetic efficiency (b, c) after 18 weeks of treatment. (d, f) Oxidation rates of pyruvate, α‐ketoglutarate, and succinate‐rotenone (used to assess Complex II–V capacity) were not significantly altered between groups, suggesting adequate function of the TCA cycle and electron transport chain. Panels (a–f): *N* = 9/group.

Isolated cardiac mitochondria from both fat‐fed groups, regardless of hypertension status, exhibited a 35% increase in State 3 oxidation capacity of the long‐chain fatty acid substrate palmitoyl‐carnitine (*p* < 0.001). The isolated mitochondrial ATP production capacity of fat‐fed mice provided palmitoyl‐carnitine was increased in proportion with oxygen consumption, resulting in no net change in isolated mitochondrial fatty‐acid bioenergetic efficiency assessed by ATP/O ratio (Figure 3a–c). Oxidation rates of pyruvate, α‐ketoglutarate, and succinate‐rotenone (used to assess Complex II–V capacity) were not significantly altered between groups (Figure 3d–f), suggesting normal function of the tricarboxylic acid cycle and the electron transport chain. Thus, isolated cardiac mitochondria from obese mice, regardless of hypertension status, exhibited only an increased capacity for mitochondrial fatty‐acid oxidation.

**FIGURE 4:**
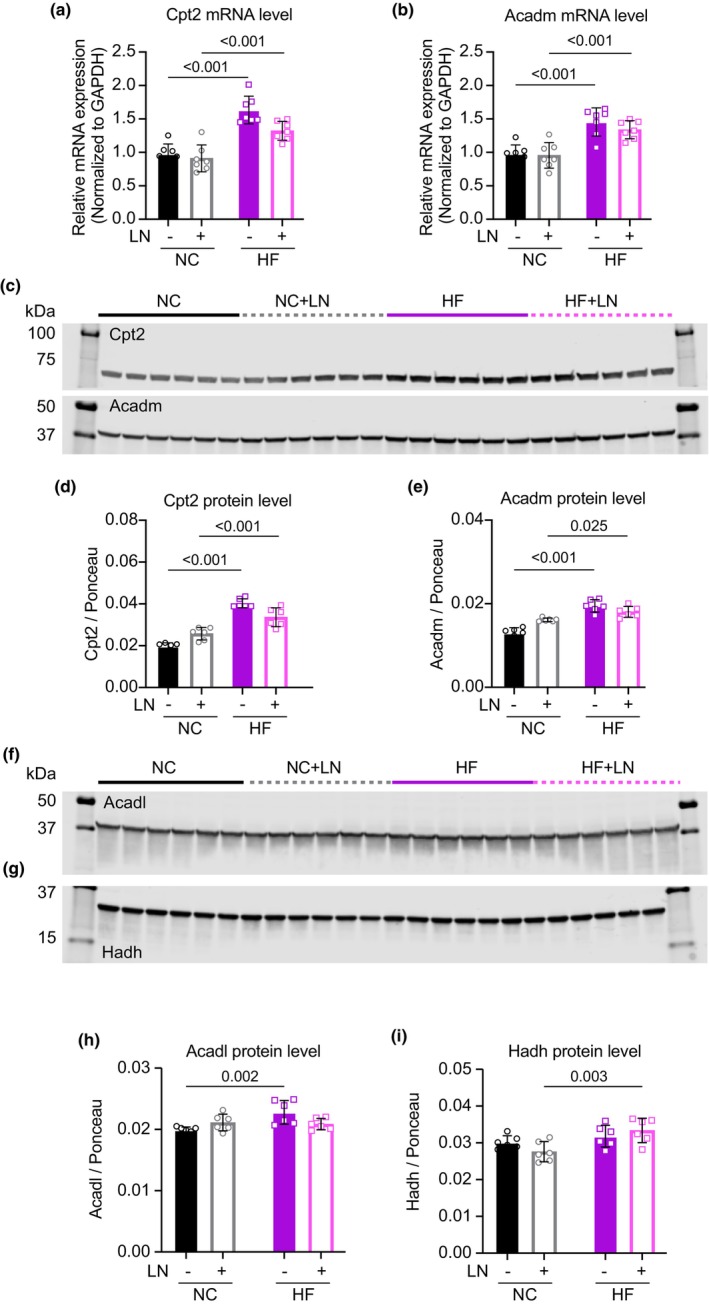
Left‐ventricular tissue homogenates from fat‐fed mice revealed significantly elevated transcript (a, b) and protein levels (c–i) of transporters and enzymes centrally involved in fatty‐acid oxidation. See Figure S3 for corresponding Ponceau stains used for protein quantification. All panels: *N* = 6–7/group.

Gene expression and protein immunoblotting analyses revealed significant increases in mRNA transcripts and protein levels of mitochondrial fatty acid transporters and beta‐oxidation enzymes in fat‐fed mice regardless of hypertension status. These proteins included carnitine palmitoyltransferase 2 (Cpt2), the inner membrane transporter responsible for mitochondrial fatty acid import, and medium‐chain acyl‐CoA dehydrogenase (Acadm), an enzyme involved directly in beta‐oxidation; both mRNA transcripts and protein levels were significantly and similarly elevated in fat‐fed groups regardless of LN administration (*p* < 0.005; Figure 4a–e). Protein levels of long‐chain acyl‐CoA dehydrogenase (Acadl) and hydroxyacyl‐coenzyme A dehydrogenase (Hadh), two additional enzymes directly involved in mitochondrial fatty‐acid oxidation, were also elevated with high fat feeding (*p* < 0.01; Figure 4f–i). Immunoblots for phosphorylation of NFATc1 and Erk1/2 did not show statistically significant differences between groups (Figure S2). All Ponceau stains used for protein quantification are shown in Figure S3.

## 4 DISCUSSION

The aim of this study was to elucidate the contribution of the respective pathophysiological drivers of the HFpEF symptom suite. By studying the independent and concomitant consequences of obesity‐driven systemic metabolic dysregulation and hypertension on cardiac structure and function, we revealed the causative drivers of cardiac functional, structural, and bioenergetic remodeling in obese normotensive, lean hypertensive, and obese hypertensive adult male mice presenting with HFpEF. Briefly, we found that hypertensive mice developed diastolic dysfunction and cardiac hypertrophy regardless of obesity status and that obese, glucose‐intolerant mice exhibited altered cardiac mitochondrial metabolism independent of hypertension status.

We found that independent of diet, obesity status, and systemic metabolic changes, both groups of hypertensive mice developed the same extent of cardiac hypertrophy as evidenced by increased heart weights normalized to tibia lengths, heart weights normalized to fat‐free mass, increased left‐ventricular mass and wall thickness, and increased average cardiomyocyte cross‐sectional area. Consistent with this trend, we found that both lean and obese hypertensive mice developed diastolic dysfunction. Significantly elevated mitral inflow velocity ratios (E/A ratio), as well as mitral annulus tissue velocity ratios (E/e' ratio) consistent with grade 3 diastolic function suggest increased early diastolic filling pressure and impaired ventricular relaxation in all chronically hypertensive mice. This observation suggests a direct role for peripheral vascular resistance in inducing cardiac hypertrophy and diastolic dysfunction in HFpEF. Our immunoblots for NFATc1 and Erk1/2 phosphorylation, two proteins involved in hypertrophic signaling, did not show statistically significant changes that might explain the molecular underpinnings of the observed pressure‐induced hypertrophic response; however, previous literature indicates that activation of these pathways is acute and transient (Crabtree & Olson, 2002; Molkentin, 2004; Zou et al., 2001). As this study design was contingent upon the development of phenotypic outcomes, we were limited in our ability to fully elucidate the early, transient hypertrophic growth signaling pathways involved in this model. Additionally, we note that obese normotensive mice also exhibited mild cardiac hypertrophy (increased LV mass and heart weight to tibia length), albeit to a much less significant extent than both hypertensive groups; thus, we must qualify that hypertension *predominantly* rather than *independently* drives cardiac hypertrophy in this model.

On the other hand, the systemic metabolic dysregulation associated with obesity, regardless of hypertension status, was clearly associated with altered cardiac mitochondrial metabolism, which we found occurred in parallel with, not as a cause or consequence of, cardiac hypertrophic remodeling. We found that isolated left‐ventricular mitochondria from fat‐fed mice exhibited an increased capacity to import and oxidize fatty acids while maintaining coupled ATP/O ratios with this substrate that is, exhibiting no deficit or advantage in net oxidative efficiency. No differences in mitochondrial bioenergetics were detected between obese hypertensive and obese normotensive mice, suggesting that during this early phase of the HFpEF cascade, cardiac hypertrophy and diastolic dysfunction do not impact the energetic capacity of the heart. Our measurements of isolated mitochondrial oxidative capacity were substantiated on a molecular level by gene expression analysis and Western blots showing that fat‐fed mice exhibit increased mRNA transcript and protein levels of a transporter and enzymes central to mitochondrial fatty‐acid oxidation; again, no differences in mitochondrial fatty‐acid oxidation transcript or protein levels were detected between obese hypertensive and obese normotensive mice. This is consistent with the fact that HFpEF hearts maintain contractile systolic function despite increased peripheral resistance. In fact, the mild hypercontractile phenotype of the HFLN+ group may well be attributable to increased heart muscle mass paired with increased capacity to oxidize and produce energy from fatty acids. In line with recent studies, we speculate that reduced cardiomyocyte glucose uptake due to insulin resistance concomitant with increased availability of free fatty acids may drive the increase in fatty acid oxidation capacity observed in obese HFpEF mice (Guven et al., 2024; Sun et al., 2024).

There remains considerable disagreement in the current literature regarding cardiac fuel utilization in HFpEF both in humans and with the use of the HFLN+ mouse model. A subsequent study from the group that originally developed this model reported a decrease in both pyruvate and palmitoyl‐carnitine oxidation capacity in isolated mitochondria and permeabilized adult mouse ventricular myocytes (Tong et al., 2021). Similarly, a recent study in humans reported decreased levels of medium‐ and long‐chain acylcarnitines in endomyocardial biopsies from human HFpEF donors compared to controls (Hahn et al., 2023), which the authors suggest indicate lower fatty acid oxidation rates similar to those observed in biopsies from HFrEF donors. This study also reported decreased utilization of alternative fuels such as glucose, ketones, branched‐chain amino acids, and tricarboxylic acid cycle intermediates. This broad, pan‐substrate deficit in cardiac fuel utilization is difficult to rectify with the maintenance of cardiac contractile function, as HFpEF patients in that study exhibited mean left‐ventricular ejection fractions of 65%, identical to controls, while HFrEF hearts had mean ejection fractions of only 18%, suggesting that fuel deficits may not limit contractile function in the HFpEF patients in that study.

In contrast, others have reported increased mitochondrial utilization of fatty acids in HFpEF hearts, which the authors suggest serves to maintain energy sufficiency for cardiac contraction and systolic function in the face of decreased intracellular glucose availability and attenuated pyruvate oxidation capacity (Guven et al., 2024; Sun et al., 2024). These studies used the same two‐hit mouse model and assessed substrate fates in isolated working hearts perfused with radiolabeled substrates and obtained similar results to the current study, which suggest that cardiac metabolic changes in HFpEF are primarily driven by obesity, with an increase in cardiac fatty acid utilization. In the present study, this observation was substantiated by increased isolated mitochondrial fatty‐acid oxidation capacity, as well as increased mRNA transcripts and protein levels of specific enzymes and transporters centrally involved in mitochondrial fatty‐acid oxidation.

In summary, all chronically hypertensive adult male mice, regardless of obesity status, presented with whole‐heart and cardiomyocyte hypertrophy, grade 3 diastolic dysfunction, and preserved systolic function. 18 weeks of chronic high‐fat feeding regardless of hypertension status resulted in an increased capacity of myocardial isolated mitochondria to oxidize and produce ATP from fatty‐acid substrates without impairing mitochondrial efficiency, and this was substantiated by increased fatty‐acid oxidation transcripts and protein levels. Taken together, our results suggests that the structural and bioenergetic HFpEF symptoms in this two‐hit model likely occur independently and additively as direct results of each of the two hits. The results of this study help to clarify the early pathogenic interplay between myocardial structural remodeling and cardiac bioenergetics in the context of HFpEF, providing nuanced mechanistic insight that may aid in the development of more precisely targeted therapeutics.

## AUTHOR CONTRIBUTIONS

**Benjamin Werbner:** conceived and designed research, performed experiments, analyzed data, interpreted results of experiments, prepared figures, drafted manuscript, edited and revised manuscript, and approved final version of manuscript. **Sophie L. Stephens:** performed experiments, analyzed data, interpreted results of experiments, prepared figures, and approved final version of manuscript. **Deborah Stuart:** performed experiments and approved final version of manuscript. **Travis M. Hotchkiss:** performed experiments and approved final version of manuscript. **Jonathan Chapman:** performed experiments and approved final version of manuscript. **Katsuhiko Funai:** interpreted results of experiments and approved final version of manuscript. **Nirupama Ramkumar:** interpreted results of experiments and approved final version of manuscript. **Sihem Boudina:** conceived and designed research, interpreted results of experiments, edited and revised manuscript, and approved final version of manuscript.

## FUNDING INFORMATION

This work was supported by the National Heart, Lung and Blood Institute (NHLBI) grants R01HL149870 and R01HL167866 (Boudina) and the National Institute of Diabetes and Digestive and Kidney Diseases (NIDDK) grants R01DK128819 (Boudina), 2T32DK110966–06 (Werbner), and T35DK103596 (Chapman and Hotchkiss).

## CONFLICT OF INTEREST STATEMENT

The authors declare no conflicts of interest.

## ETHICS STATEMENT

The study was approved by the University of Utah Animal Care and Use Commitee.

## Supporting information


Data S1.
