# Supplementary material for: Hypertension and obesity independently drive hypertrophy and alter mitochondrial metabolism in a mouse model of heart failure with preserved ejection fraction
Source: Physiol Rep. 2024 Sep 27;12(18):e70072. doi: 10.14814/phy2.70072 (PMC11427896; doi:10.14814/phy2.70072)
Supplement: Supplementary file 1 — Data S1. [file PHY2-12-e70072-s001.zip › PHYSREP-2024-03-167-T-f05-z-.docx]

**Title**

Hypertension and obesity independently drive hypertrophy and alter mitochondrial metabolism in a mouse model of heart failure with preserved ejection fraction

**Authors**

Benjamin Werbner, Sophie L. Stephens, Deborah Stuart, Travis M. Hotchkiss, Jonathan Chapman, Katsuhiko Funai, Nirupama Ramkumar, Sihem Boudina

**Supplemental Methods**

*Body composition and systemic metabolic phenotyping*

Body composition was assessed at the terminal endpoint (18 weeks of treatment; 30 weeks of age) via nuclear magnetic resonance (Bruker Mini-Spec). Fasting glucose levels were assessed after 6 hours of fasting and measured via tail snip with a handheld glucometer (Bayer Contour Next), followed by assessment of glucose homeostasis kinetics by intraperitoneal glucose administration (2 g/kg body weight) following the protocol provided by Benedé-Ubieto et al, 2023. On a separate occasion, fasting plasma insulin levels were assessed by collecting blood from the tail vein after 6 hours fasting; glucose stimulated insulin levels were assessed by collecting blood from the tail vein 30 minutes after intraperitoneal glucose administration with 2g/kg body weight. Plasma was isolated from whole blood and insulin levels were assessed using the Ultra Sensitive Mouse Insulin ELISA Kit from Crystal Chem.

*Histology*

Care was taken to ensure that technical replicates were representative of their respective sections, each image was as fully populated with cardiomyocytes as possible, and that cardiomyocyte cross-sectional orientation was consistently in the transverse plane. A custom script was written in Matlab to automate the segmentation and area quantification of approximately 50 cardiomyocytes per image; this algorithm was validated against a subset of manually quantified images (R^2^ = 0.869 between automated and manual area quantification).

*Western blotting*

Flash-frozen left-ventricular sections were homogenized in cold T-PER buffer supplemented with the Halt protease inhibitor cocktail (Thermo Fisher Scientific). Soluble proteins were isolated from the homogenate and normalized to a total protein concentration of 3 ug/uL with Laemmli buffer after protein quantification using the Pierce BCA assay kit. 45 ug of total protein was loaded into each well of a 26-well BioRad 4-15% Criterion TGX Precast Gel (n=6/group) and gel electrophoresis was run at 100V for 90 minutes. Proteins were transferred onto a 0.2 um nitrocellulose membrane via the semi-dry method using the BioRad TransBlot Turbo kit. Ponceau stain was applied to each complete membrane for 10 minutes before imaging with an Invitrogen iBright CL1500 for subsequent quantification of total protein per lane, assessed as the background-corrected intensity between 15-100 kDa. Membranes were cut horizontally based on the included Precision Plus Protein Dual Xtra Standard ladder at most twice to allow for simultaneous quantification of multiple proteins on the same membrane, as all of the primary antibodies used in this study were raised in same host species (rabbit). After 1 hour of blocking in 5% nonfat milk in Tris-buffered saline supplemented with 0.1% Tween-20 (TBST), each membrane section was incubated overnight at 4°C in 3% nonfat milk TBST supplemented respectively with one of the following antibodies at concentrations ranging from 1:1000 to 1:10,000— carnitine palmitoyltransferase 2 (Cpt2; Abcam ab181114), medium-chain acyl-CoA dehydrogenase (Acadm; Abcam ab92461), long-chain acyl-CoA dehydrogenase (Acadl; Abcam ab129711), hydroxyacyl-Coenzyme A dehydrogenase (Hadh; Abcam ab154088), pyruvate dehydrogenase kinase 4 (Pdk4; Abcam ab214938), phospho-(Ser172) nuclear factor of activated T cells (p-NFATc1; ThermoFisher PA5-64696), phospho-(Thr202/Tyr204) p44/42 mitogen activated protein kinase/extracellular signal-regulated kinase 1/2) (p-ERK; CST #9101), or total ERK1/2 (CST #4695). Subsequently, membrane sections were washed thoroughly with TBST and incubated with 5% nonfat milk TBST supplemented with LI-COR IRDye 800CW Goat anti-Rabbit IgG secondary antibody for 1 hour at room temperature. Membrane sections were washed thoroughly with TBS, imaged using the LI-COR Odyssey CLx Infrared Imaging System, and quantified densitometrically using LI-COR Image Studio software. Quantified Ponceau staining of the same membrane between 15-75 kDa was used as a normalizer for all immunoblotted proteins.

*Gene expression analysis (quantitative reverse transcription polymerase chain reaction)*

Tissues were homogenized in 1 mL Trizol (Thermo Fisher Scientific). Homogenate was centrifuged at 13,000 g for 10 minutes. The supernatant was removed and mixed with an equal volume of 100% EtOH. Total RNA from ventricular tissue was isolated using Direct-zol RNA MiniPrep (Zymo Research). Reverse transcription to cDNA was conducted using the iScript Reverse Transcription Supermix kit (Bio-Rad) per the manufacturer’s instructions. SYBR Green-based real-time PCR was perfomed using a LightCylcer 480 Real-Time PCR System (Roche). The house keeping gene Gapdh was used as an internal control for cDNA quantification and normalization using the delta-delta-CT method.

*Statistical analysis*

In order to estimate the required sample sizes to achieve 80% statistical power, an *a priori* power analysis was conducted in G*Power (Heinrich Heine University Düsseldorf) using previously published data from studies applying the two-hit HFpEF model, as well as internal preliminary data, to estimate effect sizes [Faul et al, 2007]. Unless otherwise noted, all group measurements were analyzed for statistically significant differences by first applying a two-way analysis of variance (ANOVA) in GraphPad Prism, using diet (NC or HF) and drug treatment (H2O or L-NAME) as the two factors. Sidak’s multiple comparisons post-hoc analysis was conducted to determine pairwise significant differences between groups only for the factors found significant in the two-way ANOVA (*p* < 0.05). All *p*-values from pairwise comparisons are reported as multiplicity-adjusted *p*-values. Graphically, differences between bars annotated with ****, ***, **, or * represent pairwise differences at significance levels *p* < 0.001, 0.005, 0.01, and 0.05 respectively. For graphs with more than four statistically significant differences, letters were used to indicate pairwise differences between groups for clarity, where groups not sharing a letter are statistically different at the level indicated in the figure legend.

**Supplemental Figure Legends**

Figure S1: Fat-fed mice exhibited significant increases in body weight, fat mass, fasting blood glucose, and fasting plasma insulin, as well as delayed glucose homeostasis kinetics after 18 weeks of treatment. Interestingly, chronic treatment with the pro-hypertensive nitric oxide synthase inhibitor nitro-L-arginine methylester (LN) blunted the change in glucose homeostasis kinetics observed in fat-fed mice independent of insulin levels. Panels A, C, E, F: n=15/group; B, D, G: n=5/group. Pairwise significant differences (**** p < 0.001) were determined with Sidak’s multiple comparison post-hoc analysis performed only on factors determined to be significant (p < 0.05) via two-way ANOVA. In panel F, groups not sharing letters are significantly different (p < 0.001).

Figure S2: Immunoblotting did not indicate significant differences in the phosphorylation states of NFATc1 or Erk1/2 between any groups. n=3-6/group.

Figure S3: Ponceau stains used for quantification of all immunoblotted proteins. Corresponding figures are indicated for each membrane. n=3-6/group.
