# Supplementary material for: Hypertension and obesity independently drive hypertrophy and alter mitochondrial metabolism in a mouse model of heart failure with preserved ejection fraction
Source: Physiol Rep. 2024 Sep 27;12(18):e70072. doi: 10.14814/phy2.70072 (PMC11427896; doi:10.14814/phy2.70072)

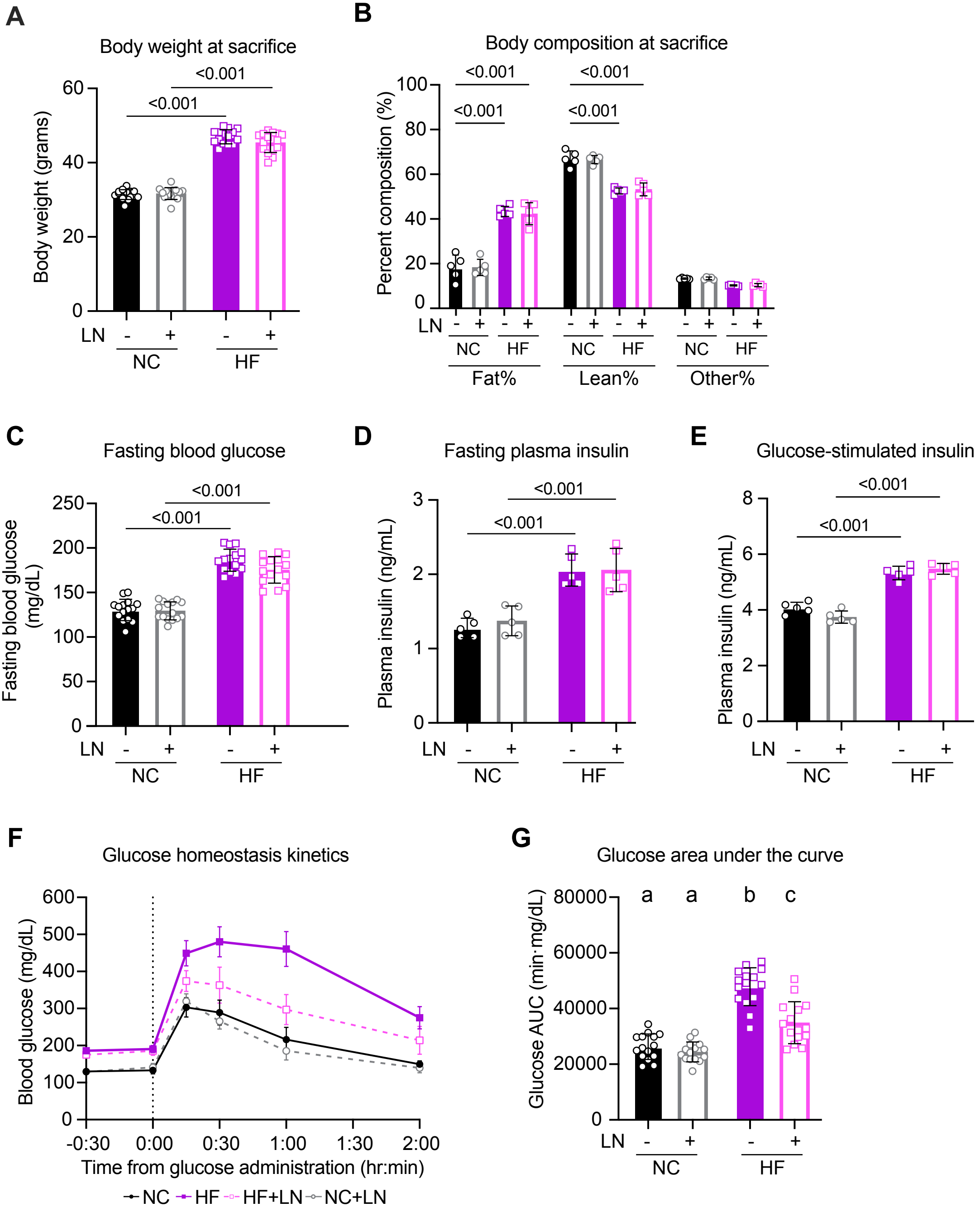
A
Body weight at sacrifice
<0.001
<0.001
Body weight (grams)
60
40
20
0
LN
-
+
-
+
NC
HF
B
Body composition at sacrifice
<0.001
<0.001
<0.001
<0.001
Percent composition (%)
100
80
60
40
20
0
LN
NC
HF
NC
HF
NC
HF
Fat%
Lean%
Other%
C
Fasting blood glucose
<0.001
<0.001
Fasting blood glucose (mg/dL)
250
200
150
100
50
0
LN
NC
HF
D
Fasting plasma insulin
<0.001
<0.001
Plasma insulin (ng/mL)
3
2
1
0
LN
NC
HF
E
Glucose-stimulated insulin
<0.001
<0.001
Plasma insulin (ng/mL)
8
6
4
2
0
LN
NC
HF
F
Glucose homeostasis kinetics
Blood glucose (mg/dL)
600
500
400
300
200
100
0
-0:30
0:00
0:30
1:00
1:30
2:00
Time from glucose administration (hr:min)
NC
HF
HF+LN
NC+LN
G
Glucose area under the curve
a
a
b
c
Glucose AUC (min·mg/dL)
80000
60000
40000
20000
0
LN
NC
HF

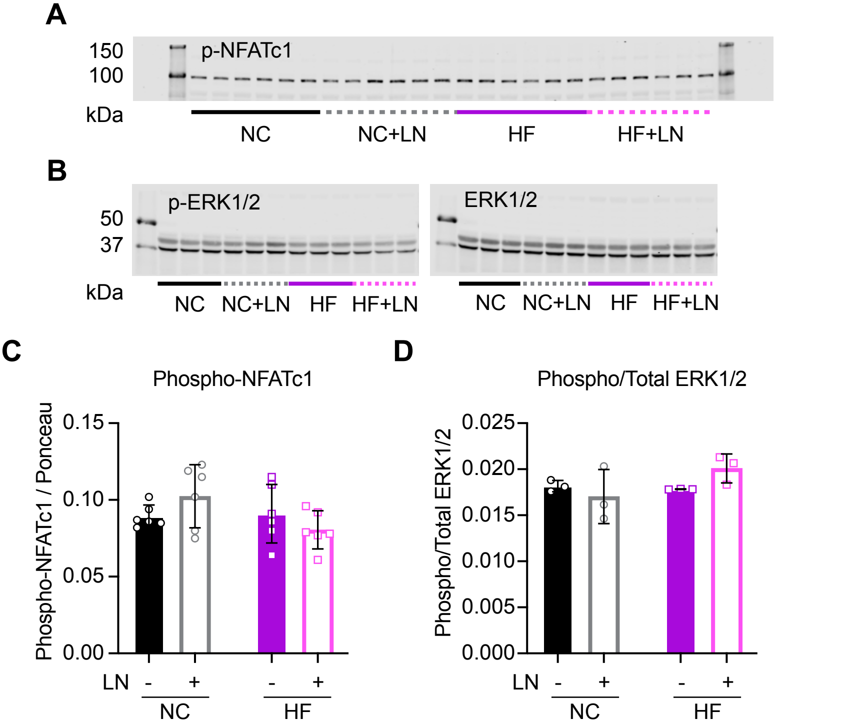

A
p-NFATc1
150
100
kDa
NC
NC+LN
HF
HF+LN
B
p-ERK1/2
ERK1/2
50
37
kDa
NC
NC+LN
HF
HF+LN
C
Phospho-NFATc1
Phospho-NFATc1 / Ponceau
0.15
0.10
0.05
0.00
LN
-
+
NC
HF
D
Phospho/Total ERK1/2
Phospho/Total ERK1/2
0.025
0.020
0.015
0.010
0.005
0.000

A

Cpt2 and Acadm Ponceau
(Corresponding Figure 4C)

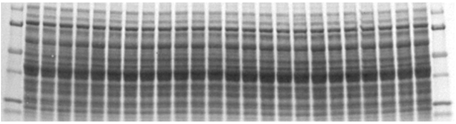

B

Acadl Ponceau
(Corresponding Figure 4F)

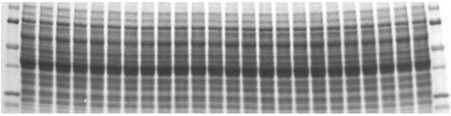

C

Hadh Ponceau
(Corresponding Figure 4G)

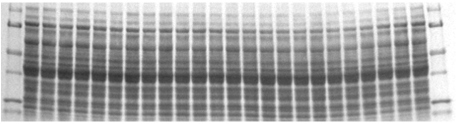

D

Phospho-NFATc1 Ponceau
(Corresponding Figure S2A)

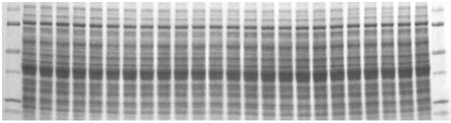

E

Total- and Phospho-ERK1/2 Ponceaus
(Corresponding Figure S2B)

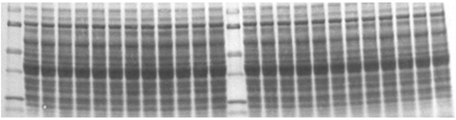

Supplement: Supplementary file 1 — Data S1. [file PHY2-12-e70072-s001.zip › PHYSREP-2024-03-167-T-f06-z-.pdf]
